# Commentary: Cerebral Lateralization is Protective in the Very Prematurely Born

**DOI:** 10.3389/fpsyg.2016.00903

**Published:** 2016-06-16

**Authors:** Deborah J. Serrien

**Affiliations:** School of Psychology, University of NottinghamNottingham, UK

**Keywords:** preterm, resting state, brain maturation, functional MRI, language development

The cerebral hemispheres of the human brain have unique functional properties of information processing; an asymmetry that captures cerebral lateralization. Among cognitive functions, language is one of the most lateralized with left-sided dominance for the production and processing of language. Previous work has shown that the foundation of language circuitry emerges in the late second and third trimesters in developing fetuses, supported by a genetically driven phase of development (Pinel et al., 2012; Thomason et al., 2014). The significance of this developmental period in the neonate is underlined from research in individuals born preterm who show altered brain development, and who are at risk for developmental delays and impairments in neuropsychological performance such as deficits in language processing (Poggi Davis et al., 2011; Kwon et al., 2014). Due to advanced MRI techniques, new and detailed insights can be obtained from changes to the typical developmental pattern of cerebral lateralization.

Scheinost et al. (2015) examined cerebral lateralization in adolescents who were born very preterm (VPT, with a mean gestational age of < 30 weeks) in order to test the hypothesis that preterm birth remodels the intrinsic functional infrastructure of the brain. The authors made use of resting-state functional magnetic resonance imaging (rs-fMRI) alongside a novel voxel-based approach that estimates connectivity lateralization without the requirement to predefine regions of interest. Measuring brain connectivity at rest provides a powerful method for unraveling the neural correlates of the cognitive systems they support. A correlation analysis between the connectivity lateralization patterns and language performance—the Peabody Picture Vocabulary Test-Revised—was calculated for assessing an association between brain function and behavior.

The data of the VPT group showed clusters of significant correlations between the language task and connectivity lateralization in left-sided frontal-temporal language regions and their right hemisphere homologs. In particular, a positive correlation was noted between the language task and connectivity lateralization in the left-sided areas whereas the correlation was negative between the language task and the right-sided regions. Most successful on the language task were the VPT individuals with the strongest lateralization in the left hemisphere and lowest lateralization in the right hemisphere, underlining the strength of the typical left-sided pattern alongside a rebalancing of activity within the existing neural circuitry.

A further observation for the VPT individuals was that the right-sided language homolog regions highly correlated with one other, with the right angular gyrus operating as a functional integration hub that supported not only the intrahemispheric connections but also the interhemispheric connections with the left-sided language regions. This finding highlights the presence of a bihemispheric network for language in adolescents born VPT. From developmental research, it has been established that the language network of children is regulated by interhemispheric circuitry, which progresses into an adult's language network that is controlled by a frontal-to-temporal circuit lateralized to the left hemisphere. Therefore, a shift from interhemispheric to intrahemispheric control is part of the maturation process, accompanied by a balanced increase in functional connectivity within the left hemisphere and a disconnection from contralateral hemispheric activity (Friederici et al., 2011).

The alterations in connectivity lateralization between the language centers in the VPT individuals indicate a neural reorganization that modifies the coordinated activity between brain regions. The observed changes in connectivity could reflect a compensatory mechanism within the right hemisphere (Wilke et al., 2014). Previous research has pointed out that the recruitment of a language circuit with bilateral pathways can be triggered by events that alter pre-determined phases in prenatal development (Myers et al., 2010). Alternatively, it is possible that delays in cerebral maturation in the VPT individuals (Gozzo et al., 2009) enabled to preserve the right-hemispheric connection. Based on these findings, it remains to be determined whether lateralization changes of functional systems interact with one another such as language and visuospatial functions that have their dominant representations in opposite hemispheres in adulthood (Cai et al., 2013).

Notwithstanding that the VPT individuals showed significant correlations between the language scores and connectivity lateralization, they performed comparably to the full term control individuals for the language task. Furthermore, no significant effect of handedness was observed, although it is noteworthy that the number of right-handers was lower in the VPT (80%) as compared to the full term (96%) group. This observation is relevant considering that increased non-right handedness is noted in preterm individuals (Domellöf et al., 2011), and that the developmental trajectory of hand preference significantly predicts the developmental progression of language (Michel et al., 2013). Overall, handedness is a factor that influences organizational mechanisms of behavior and guides intrahemispheric and interhemispheric pathways in distinct ways (Reid and Serrien, 2012).

Taken together, the data from Scheinost et al. (2015) underscore the important premise that fundamental alterations in cerebral lateralisation occur due to disruption of critical time periods in early development. Preterm birth perturbs cerebral development at least up to adolescence, with changes in the connectivity patterns that support cognitive functions. The current findings further highlight the significance of network analysis for revealing principles of brain maturation and for uncovering shifts in connectivity lateralization that could be used as predictors for developmental progression and outcomes in order to promote targeted interventions.

## Author contributions

The author confirms being the sole contributor of this work and approved it for publication.

### Conflict of interest statement

The author declares that the research was conducted in the absence of any commercial or financial relationships that could be construed as a potential conflict of interest.
